# Prodepth: Predict Residue Depth by Support Vector Regression Approach from Protein Sequences Only

**DOI:** 10.1371/journal.pone.0007072

**Published:** 2009-09-17

**Authors:** Jiangning Song, Hao Tan, Khalid Mahmood, Ruby H. P. Law, Ashley M. Buckle, Geoffrey I. Webb, Tatsuya Akutsu, James C. Whisstock

**Affiliations:** 1 Department of Biochemistry and Molecular Biology, Monash University, Clayton, Melbourne, Victoria, Australia; 2 Bioinformatics Center, Institute for Chemical Research, Kyoto University, Gokasho, Uji, Kyoto, Japan; 3 Faculty of Information Technology, Monash University, Clayton, Melbourne, Victoria, Australia; 4 ARC Centre of Excellence for Structural and Functional Microbial Genomics, Monash University, Clayton, Melbourne, Victoria, Australia; Indiana University School of Medicine, United States of America

## Abstract

Residue depth (RD) is a solvent exposure measure that complements the information provided by conventional accessible surface area (ASA) and describes to what extent a residue is buried in the protein structure space. Previous studies have established that RD is correlated with several protein properties, such as protein stability, residue conservation and amino acid types. Accurate prediction of RD has many potentially important applications in the field of structural bioinformatics, for example, facilitating the identification of functionally important residues, or residues in the folding nucleus, or enzyme active sites from sequence information. In this work, we introduce an efficient approach that uses support vector regression to quantify the relationship between RD and protein sequence. We systematically investigated eight different sequence encoding schemes including both local and global sequence characteristics and examined their respective prediction performances. For the objective evaluation of our approach, we used 5-fold cross-validation to assess the prediction accuracies and showed that the overall best performance could be achieved with a correlation coefficient (CC) of 0.71 between the observed and predicted RD values and a root mean square error (RMSE) of 1.74, after incorporating the relevant multiple sequence features. The results suggest that residue depth could be reliably predicted solely from protein primary sequences: local sequence environments are the major determinants, while global sequence features could influence the prediction performance marginally. We highlight two examples as a comparison in order to illustrate the applicability of this approach. We also discuss the potential implications of this new structural parameter in the field of protein structure prediction and homology modeling. This method might prove to be a powerful tool for sequence analysis.

## Introduction

In order to perform their biological function, most proteins naturally fold into a defined native three-dimensional structure. Given the globular nature of the majority of proteins, residues can generally be classified as buried or solvent exposed. Solvent exposed residues commonly perform key roles such as mediating protein-protein interactions as well as influencing protein stability, whereas buried residues are often thought of as important determinants of protein folding [1]. Given a protein structure, it is relatively straightforward to broadly identify a residue as buried or exposed. However, a more precise recognition of the burial status or the burial degree of a residue is often useful to more closely understand its functional role [2], [3], [4], [5], [6], which is not only necessary for our deep understanding of the sequence-structure-function relationship and protein folding mechanism [7], [8], [9], [10], but also helpful for predicting protein structural and functional properties [11], as well as protein engineering and de novo drug design [8], [11], [12]. Further, these data are becoming increasingly useful in understanding how proteins fold, and from a disease perspective, misfold. Finally, predicted solvent accessibility information has been proved useful in prediction of protein flexibility [13], natively unstructured regions [14], [15], DNA-binding site [16] and protein interaction hot-spots from sequences [17].

Conventionally, residue burial is quantified by an exposure measure called solvent accessible surface area (ASA), which is a structural descriptor that has been widely used in the analysis of protein structure and function in the last three decades [2]–[6]. Typically, ASA is calculated using the ‘rolling ball’ algorithm that was developed by Shrake and Rupley [18], which uses a sphere radius to probe the surface of the molecule. It is worthy to point out that the chosen probe radius has an effect on the observed ASA values: the smaller the sphere radius, the larger the calculated ASA. A typical radius value of 1.4 Å is often adopted in the literature, which approximates the radius of a water molecule. The use of ASA is particularly effective for the analysis of exposed surface residues. However, beneath the surface of a protein, ASA fails to describe to what extent a residue is buried. Accordingly, ASA does not have the capacity to readily distinguish a substantially buried residue that is nevertheless close to the surface from a residue that is truly completely deep in the hydrophobic core; for both cases the ASA value would be zero or near zero. For this purpose a complementary descriptor, residue depth (RD), can be calculated [7]–[10].

RD measures the distance between the residue of interest and its nearest neighboring water molecule or protein surface [7]–[9], [19], [20], providing important information about the extent to which the residue is buried in the protein structure. Previous studies have established that RD is correlated with several protein properties, such as protein stability, residue conservation and amino acid types [7]. Other RD-derived measures have also been proposed to help analyze protein structure and function: one example is pocket depth [21] and another is travel depth [22]. Due to its advantages, RD has attracted increasing attention in recent years. RD has been shown to correlate with other measures and has been applied recently to effectively improve the accuracy of protein fold recognition [23], [24], [25]. In addition, RD could also complement the information provided by traditional measures such as ASA [2]–[5], solvent accessibility [6], [11], half-sphere exposure [26], [27], recursive convex hull (RCH) [28] and residue contact number (CN) [29], [30]. Hence, RD has several advantages relative to other traditional solvent exposure measures, which would have potentially important applications with respect to protein structure prediction and homology-based modeling [31], [32].

Currently, predictions in regards to whether a residue is exposed or buried are used in a wide variety of protein structure prediction engines [6], [33]. Such prediction can provide valuable information for protein fold recognition, functional residue prediction and protein drug design. For example, experimental evidence shows that some functionally important residues need to be specifically exposed [34] or buried [35] in order to play their critical roles. Thus the ability to accurately predict RD would be anticipated to be of importance. Further, interesting questions in regards to the extent of the relationship between RD and amino acid sequence remain to be fully understood.

To date several approaches to predict RD have been published. Notably, Yuan and Wang proposed a computational framework that uses sequential evolutionary information contained in PSI-BLAST profiles and the global protein size information to quantify the relationship between RD and protein sequence [36]. As a result, their method could predict the RD distribution with the correlation coefficient of 0.65 between the observed and predicted RD values [36]. More recently, Zhang *et al*. proposed the RDpred method to predict RD values by applying several informative features such as predicted secondary structure, residue position and PSI-BLAST profile, which has achieved correlation coefficients of 0.67/0.67 between observed and predicted RD values, when evaluated using 3-fold/10-fold cross-validations [37].

In this article, we describe a sequence-based method that also uses support vector regression to quantify the RD-sequence relationship. However, we not only exploit sequence information previously used (position-specific scoring matrices in the form of PSI-BLAST profiles, predicted secondary structure and protein sequence length), but also take into account other sequence and structural features that are not used in previous studies (predicted solvent accessibility, natively unstructured regions, percentage of exposed/buried residues, percentage of secondary structure classes and percentage of ordered/disordered residues). More importantly, these additional features have been demonstrated to make a substantial contribution to the prediction performance improvement. To objectively evaluate the proposed approach, we used 5-fold cross-validation to examine the prediction accuracies and showed that the overall best performance could be achieved with a correlation coefficient of 0.71 between the observed and predicted RD values after incorporating all the relevant multiple sequence and structural features, which has significantly outperformed the previously described methods. The results suggest that RD can indeed be accurately predicted from protein primary structure only and particularly the predicted solvent accessibility information has a significant effect on the prediction performance. As an implementation of this methodology, we have developed a prediction web server Prodepth, which is freely available at http://sunflower.kuicr.kyoto-u.ac.jp/~sjn/Prodepth/.

## Results

### The skewed distribution of residue depth

**Figure 1:**
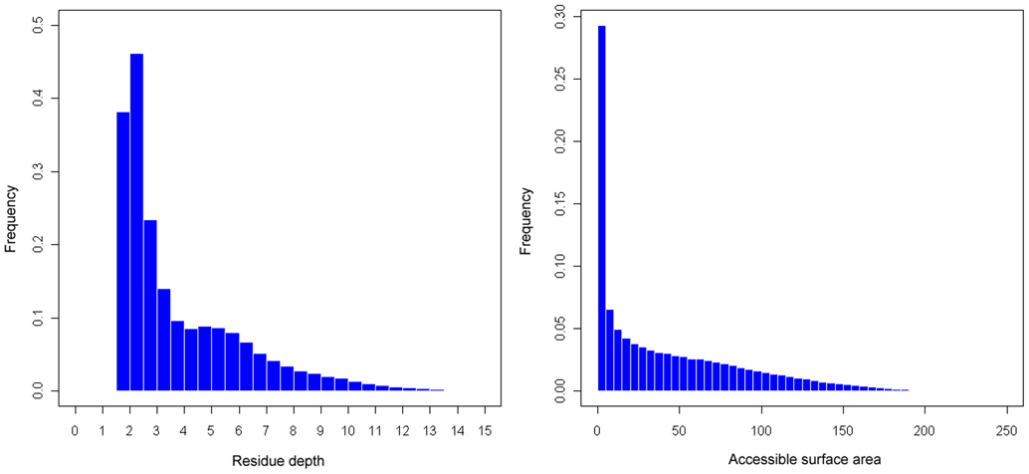
The skewed distributions of residue depth and accessible surface area for all residues based on the current dataset.

We calculated RD for all residues in our dataset with the detection sphere radius set up as 13 Å and showed their distributions in Figure 1. The results indicate that RD shows a skewed distribution. Nearly all the residues are located in the depth range from 1.2 to 10 Å, which covers 96.8% of the total residues. Furthermore, about 74.2% of the residues in the dataset are found with residue depth less than 5 Å, which means that most residues are actually located at the protein surfaces [36]. This is in sharp contrast to deeply buried residues with larger depth values (>5 Å), which only account for 25.8% in the current dataset. In addition, the mean value and standard deviation for this skewed RD distribution are 3.988 and 3.259 Å, respectively. As a contrast, the mean and standard deviation for the ASA distribution are 42.89 and 45.93 Å, respectively. The skewed RD distribution is in a similar trend as the ASA distribution (Figure 1).

**Figure 2:**
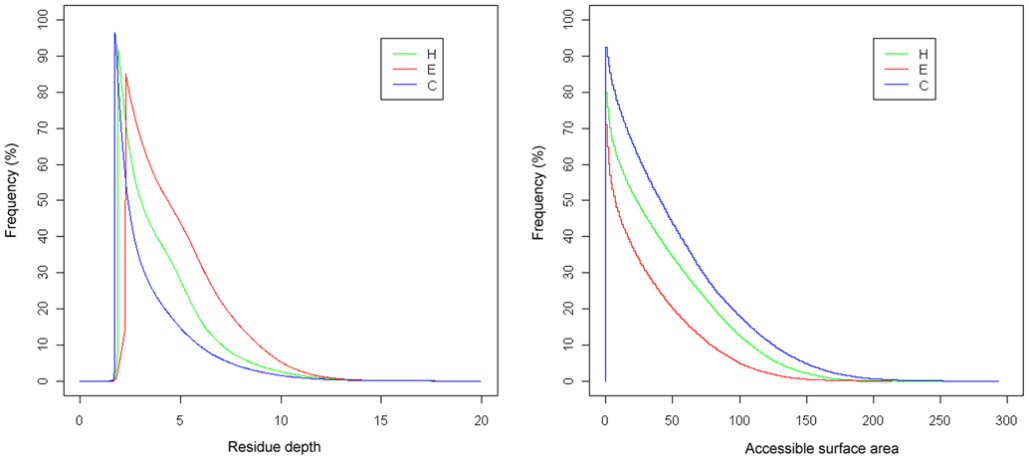
Distributions of RD and ASA according to three secondary structure classes: helix (H), strand (E) and coil (C).

We further extracted the secondary structure annotations for all residues in the current dataset using the DSSP program [38] and calculated their distributions as displayed in Figure 2, according to three secondary structures: α-helix (H), β-strand (E) and coil (C) [39]. We used the common CK mapping proposed by Chandonia and Karplus [39] to further classify the eight secondary structures assigned by DSSP into three classes: α-helix (H→H), β-strand (E→E) and other irregular or unstructured elements (all others→C). Note that in this classification all the irregular or unstructured elements are classified as coils (C). Residues with secondary structures of α-helix, β-strand and coil account for 33.2%, 21.1% and 45.7%, respectively. Their mean RD values and standard deviations are 4.16±3.40, 5.10±3.30 and 3.35±2.97 Å, respectively. It can be further observed that β-strand residues (red-color) tend to have larger residue depth values, implying that they are more deeply buried compared with other secondary structure-annotated residues. On the other hand, coiled residues are less deeply buried, as they are more frequently observed to have smaller RD values. In the case of the ASA distribution, coiled residues tend to have larger ASA values than other two secondary structures, while β-strand residues are found to have smaller ASA values (Figure 2).

**Figure 3:**
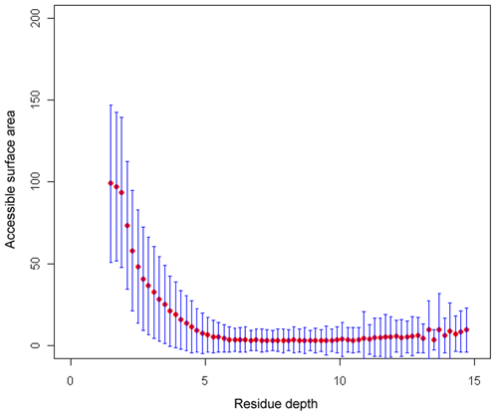
The relationship between RD and ASA. Error bars represent the standard deviations.

To investigate the interdependencies of various solvent exposure measures, we calculated the correlation coefficients between RD and other measures such as ASA, rASA, CN and B-factor (Table 1). Measure pairs that have low correlation coefficients are likely to be unrelated and can potentially provide complementary information for each other [28]. As a residue's rASA value is calculated as the normalization of its ASA using the maximum ASA for its residue type, it is easy to understand that ASA and rASA are strongly correlated with a CC of 0.92. On one hand, RD is correlated with CN (0.77). On the other hand, RD is negatively correlated with the ASA (−0.62) and rASA measure (−0.66), respectively, which is understandable as residues with smaller ASA values are inclined to be buried deeply and as a consequence they would have larger RD values. This negative correlation between RD and ASA is also clearly manifested in Figure 3. We also calculated the CC between RD and B-factor, but did not find any strong correlation between these two measures (Table 1).

**Table 1:** The correlation coefficients between residue depth and other structure-based solvent exposure measures and B-factor.

Measures	RD	ASA	rASA	CN	B-factor
RD	1.00	−0.62	−0.66	0.77	−0.44
ASA	−0.62	1.00	0.92	−0.70	0.43
rASA	−0.66	0.92	1.00	−0.75	0.46
CN	0.77	−0.70	−0.75	1.00	−0.57
B-factor	−0.44	0.43	0.46	−0.57	1.00

In addition, the negative correlation between RD and ASA indicates that they are virtually distinct characterizations of spatial environments of residues and are complementary to each other. In order to further explore the relationship between RD and ASA, we obtained the ASA values for all the residues in our dataset and computed their mean values and standard deviations. The results are shown in Figure S1, which clearly suggests that there is a negative correlation between RD and ASA measures.

### Predicting residue depth based on evolutionary profiles in the form of PSSMs

In this section, we employed the support vector regression approach to quantify the sequence-RD relationship and predict the RD values based on primary sequences information only. We aligned each protein sequence in our dataset against the NCBI nr database with three iterations to obtain the evolutionary profiles of PSI-BLAST. Then we used the sliding windows to capture the local sequence environment, that is, we included the PSSMs of w = 15 consecutive residues (upstream 7 and downstream 7 residues) as the input features into the SVR. The prediction accuracies of the built SVR models were examined using 5-fold cross-validation method to avoid the biased evaluation. Aside from the R-square, three other measures CC, RMSE and MAE were used to evaluate the prediction accuracy of the SVR approach, as adopted in other sequence-based real-value prediction studies, such as residue contact number [40], residue-wise contact order [41] and half-sphere exposure [27].

In our efforts to further improve the prediction performance, we have developed the SVR models based on up to eight different sequence encoding schemes. Unless otherwise stated, we refer to the encoding schemes based on PSI-BLAST profile, PSIPRED-predicted secondary structure, SCRATCH-predicted solvent accessibility, DISOPRED-predicted natively disordered regions, protein sequence weight and length, and all the combined sequence features, as ‘PB’, ‘PP’, ‘SC’, ‘DISO’, ‘WL’ and ‘ALL’, respectively. The prediction performance of these different sequence encoding schemes is presented in Table 2.

**Table 2:** Prediction accuracy of the SVR predictors based on eight different sequence encoding schemes that incorporate various combinations of global and local sequence features.

Sequence encoding scheme	Number of features	Number of support vectors	CC	RMSE	MAE	R square
PB	300	100134	0.64±0.02	1.90±0.10	1.41±0.08	0.456±0.02
SC	30	99021	0.65±0.03	1.86±0.11	1.34±0.10	0.474±0.03
PB+PP	345	100045	0.66±0.02	1.88±0.11	1.39±0.09	0.537±0.03
PB+SC	330	99662	0.69±0.03	1.77±0.10	1.31±0.08	0.539±0.03
PB+PP+SC	375	99682	0.70±0.03	1.76±0.11	1.30±0.09	0.540±0.03
PB+PP+SC+DISO	405	99719	0.70±0.03	1.75±0.11	1.29±0.09	0.539±0.03
PB+PP+SC+DISO+WL	407	99319	0.70±0.03	1.75±0.11	1.29±0.09	0.539±0.03
ALL	435	103631	0.71±0.03	1.74±0.10	1.28±0.08	0.541±0.03

All results were evaluated using 5-fold cross-validation method and expressed as mean±standard deviation.

In particular, the SVR model based on the encoding scheme “PB” could predict the RD distributions with a CC of 0.64 between the predicted and observed RD values, a RMSE of 1.90, and MAE (mean absolute error) of 1.41 respectively, when using evolutionary information in the form of PSSMs as input features. These results indicate that using only evolutionary information contained in the PSSMs could provide rather good RD predictions, consolidating the previous conclusions from other studies that the PSSM profiles make more important contribution to the prediction performance than the single sequences alone [13]–[17], [27], [40], [41], [42], [43], [44], [45].

### Predicted secondary structure significantly improves the prediction performance

However, the prediction performance can be further improved by incorporating other informative features, such as the predicted secondary structures. Prediction accuracy based on sequence encoding scheme ‘PB+PP’ (CC = 0.66 and RMSE = 1.88) is better than that based on ‘PB’ (CC = 0.64 and RMSE = 1.90), suggesting that incorporating the predicted secondary structure by PSIPRED could significantly boost the performance. This is also the case when we compare the prediction accuracy based on encoding schemes ‘PB+SC’ and ‘PB+PP+SC’: the accuracy of the latter is better than the former, with the CC increasing from 0.69 to 0.70 and the RMSE decreasing from 1.77 to 1.76, respectively.

The significance of the inclusion of the local sequence information in the form of predicted secondary structure on the prediction performance has been demonstrated in previous studies, such as the prediction of transmembrane protein topology [43], disulfide connectivity pattern [42], [46], half-sphere exposure [27], recursive convex hull class assignments [28], protein fold classification [47], and the twilight-zone protein structural class assignments [48], [49].

### Global sequence features improves the prediction performance marginally

Moreover, prediction accuracy can be slightly improved by taking account of protein size information, as measured by sequence length descriptor ‘L’ and molecular weight descriptor ‘W’. For the former, we calculated the mean sequence length and standard deviation for all the proteins and normalized and encoded them into SVR. For the latter, a protein's weight was simply calculated as the summation of all its amino acid residues' weights and was further normalized using the corresponding mean and SD values.

Furthermore, we included other global sequence information in the form of twenty amino acid compositions, the percentage of three predicted secondary structures, the percentage of predicted exposed/buried residues, and the percentage of natively disordered/ordered residues as well. These global sequence features, in combination with the local sequence profiles generated by PSI-BLAST, PSIPRED, SCRATCH and DISOPRED programs, constitute the termed sequence encoding scheme ‘ALL’, leading to the best prediction performance with the CC of 0.71, the RMSE of 1.74 and the MAE of 1.28.

### Predicted solvent accessibility information considerably improves the prediction performance

Noticeably, we found that the prediction accuracy could be considerably improved after incorporating the predicted solvent accessibility information generated by the SCRATCH program [50]. It is worth mentioning that using the simple encoding scheme ‘SC’ solely can lead to a prediction performance of CC = 0.65 and RMSE = 1.86, respectively, which is competitively comparable to that of ‘PB’. The improvement of prediction performance using ‘SC’ only is remarkable, considering that the SVR model based on the predicted ASA only used 30 features, compared to the PSSM profile (‘PB’) which requires 300 features (Table 2).

In comparison with the sequence encoding scheme ‘PB’ with evolutionary profiles, the SVR model based on the encoding scheme ‘PB+SC’ can achieve a prediction accuracy of CC = 0.69 and RMSE = 1.77, with the CC increased by 0.05 and RMSE decreased by 0.13, respectively, which is a considerable performance improvement. The value of MAE lowers by 0.10 and the R square value also increases from 0.456 to 0.539 accordingly. Moreover, we can draw the same conclusion by comparing the prediction accuracy of ‘PB+PP’ and ‘PB+PP+SC’ (Table 2). This finding indicates that the predicted solvent accessibility is a very important sequence feature that contributes significantly to the performance improvement when combined with other features. We also found that incorporating natively disordered regions predicted by DISOPRED improves the prediction performance further even further.

**Figure 4:**
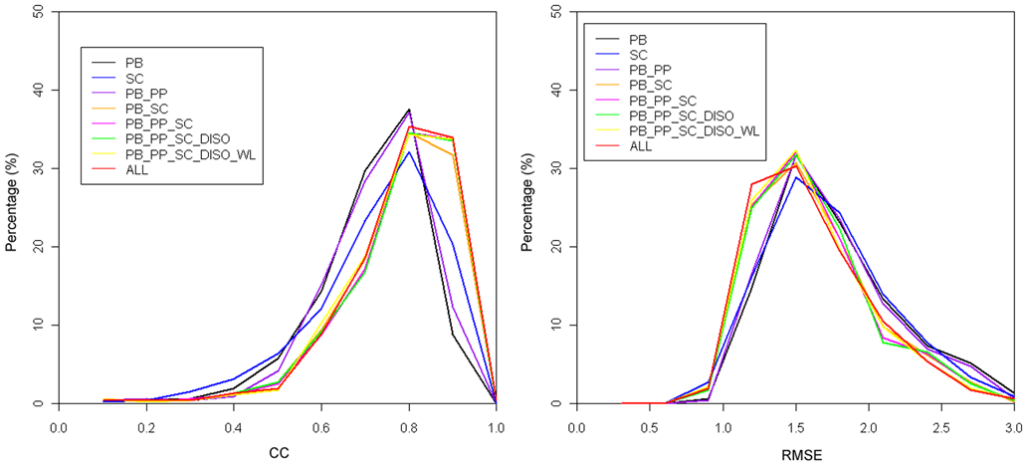
The distributions of correlation coefficients and root mean square errors based on eight different sequence encoding schemes.

The distributions of CC and RMSE based on eight different sequence encoding schemes are given in Figure 4. In particular, the peak values of CC and RMSE for all these encoding schemes are around 0.8 and 1.5, respectively, which can be regarded as the upper limits of the prediction performance. In the case of the CC distribution, we observed that five encoding schemes ‘PB_SC’, ‘PB_PP_SC’, ‘PB_PP_SC_DISO’, ‘PB_PP_SC_DISO_WL’, and ‘ALL’ are located very closely, implying that they have similar performances when evaluated by the CC measure. In the case of RMSE distribution, the leftmost curve (encoding scheme ‘ALL’) in the plot represents the best prediction method. In contrast to CC, RMSE is a more sensitive measure that clearly reflects the prediction performance improvement.

In solvent accessibility prediction, it has been a common practice to divide accessibility levels into bins and assess the prediction performance using classification measurements. As suggested by a reviewer, we classified the depth profiles of all residues in the dataset into depth bins with a separation of 3 Å and calculated the corresponding prediction accuracies of different sequence encoding schemes according to the depth bins. The results are shown in Table 3. For all the sequence encoding schemes, the prediction accuracies decrease with the increasing depth levels. For example, for the depth level 0–3.0 Å, the prediction accuracies of different encoding schemes vary from 70.2% to 74.4%, while for the depth level 9.0–12.0 Å, the accuracies fall within the range of 0–10.4%. These results indicate that the predicted depth values for deeply buried residues are less accurate and therefore they are much more difficult to predict. Use of the best encoding scheme ‘ALL’ significantly improves the prediction performance for the deeply buried residues.

**Table 3:** Prediction accuracy of different sequence encoding schemes according to the depth bins.

Model	Sequence encoding scheme	0–3.0	3.0–6.0	6.0–9.0	9.0–12.0	12.0–15.0
1	PB	70.2	63.4	26.4	5.8	0.18
2	SC	73.7	64.1	38.2	0	0
3	PB+PP	74.4	64.9	39.1	6.9	0.12
4	PB+SC	71.1	62.8	29.5	7.2	0.12
5	PB+PP+SC	74.2	64.7	39.8	8.8	0.06
6	PB+PP+SC+DISO	73.9	64.7	39.8	8.8	0.06
7	PB+PP+SC+DISO+WL	73.5	64.2	40.2	9.9	0.29
8	ALL	73.4	64.9	41.2	10.4	0.26

### Analyzing the mean absolute errors

**Figure 5:**
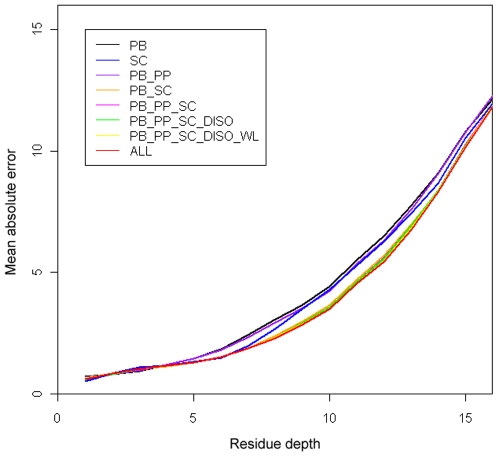
The mean absolute errors (MAEs) for residues with different RD values using eight different sequence encoding schemes.

To measure the prediction performance of residues with different depth values, we calculated the absolute errors for residues with depth values from 0–20, whose MAE distributions based on eight encoding schemes are depicted in Figure 5. For the majority of residues with varying RD values, the sequence encoding scheme ‘ALL’ (denoted by red-colored curve, which is beneath all the other seven curves in Figure 5) provides the least absolute errors, representing the best prediction method. In addition, residues with RD values ranging from 1.0 and 5.0 are predicted with lesser mean absolute errors, indicating that these data points are more adequately represented in the current dataset and hence are better predicted by the SVR approach. On the other hand, predictions for residues with larger depth values are comparatively poor. It is possible that this is due to the inadequate or under representation of deeper residues in our data.

### The predicted RD distribution according to three secondary structures

To understand the relationship between the prediction errors and secondary structures, we calculated the mean absolute errors and percentage distributions according to three secondary structures (α-helix, β-strand and coil) based on four ranges of the observed RD values, as shown in Table 4. The results revealed three points: First, the mean absolute errors will increase with the increasingly buried extent of residues. This applies to all the three secondary structures. Second, irregular coiled residues tend to be exposed, compared with other regular secondary structures. For the latter, they are more frequently observed in the protein core regions. Third, coiled residues tend to have larger MAE values, indicating that they are less accurately predicted. It might be that the under-representation of these coiled residues makes them less adequately represented when building the training SVR models.

**Table 4:** The mean absolute errors (Å) and percentage (%) distributions of three secondary structures based on four ranges of residue depth values.

RD values (Å)	α-helix (Å, %)	β-strand (Å, %)	coil (Å, %)
0–2.0	0.56, 31.7	0.73, 9.1	0.52, 59.2
2.0–2.5	0.71, 41.6	0.86, 19.1	0.64, 39.3
2.5–3.0	0.88, 42.4	1.00, 26.4	0.79, 31.2
>3.0	1.85, 42.0	1.84, 37.2	2.14, 20.8

**Figure 6:**
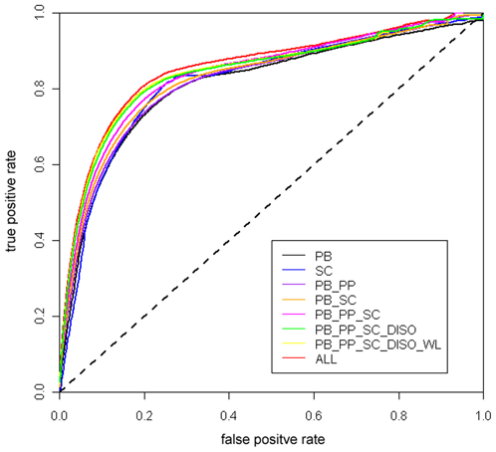
Receiver Operating Characteristics for the two-state solvent accessibility prediction based on the predicted residue depth values using eight different sequence encoding schemes. The ROC curves can be used to discriminate the classification performances of different encoding schemes. The diagonal line represents a completely random guess.

We also examined the prediction accuracy by making two-state solvent accessibility assignments, i.e. predicting whether a residue is buried or exposed based on its predicted RD values. As both the ASA and RD measures can be used to assign the buried or exposed residues, we adopted the strategy proposed by Yuan and Wang [36] and set up the RD threshold at 3.03 Å to maximize the consistency percentage (CP) to reach the best agreement between ASA and RD (See Figure S2, RD = 3.03 and ASA = 29.34 Å at the crossing point of two curves, respectively). After applying the RD threshold of 3.03 Å to discriminate the exposed (< = 3.03 Å) and buried (>3.03 Å) residues in the current dataset, we achieved prediction accuracies of 74.1% and 82.9% for exposed and buried residues, respectively, with the overall prediction accuracy of 78.2%. The prediction performance of the two-state solvent accessibility assignment can be evaluated by comparing the areas under the receiver operating characteristic (ROC) curves. As can be seen from Figure 6, the SVR model based on sequence encoding scheme ‘ALL’ surpasses all the other models, which means that this encoding scheme has better sensitivity values given any choice of specificity in contrast to other encoding schemes.

### Comparison to previous methods

The prediction performance of our approach was compared to two previous prediction schemes [36], [37], as shown in Table 5. As the prediction comparison is meaningful only provided that it is performed based on the same datasets and evaluated using the same performance evaluation measures, we implemented these two methods previously proposed and applied three measures to evaluate the prediction performance based on the current dataset. The CC of the Prodepth (CC = 0.71) is higher than that of the Yuan-Wang method (CC = 0.64) and that of the RDpred method (CC = 0.68) proposed by Zhang *et al*. One the other hand, Prodepth achieved a RMSE of 1.74 and a MAE of 1.28, respectively. The RDpred approach based on all sequence features achieved a RMSE of 1.84 and a MAE of 1.36, receptively, while for Yuan and Wang's approach a RMSE of 1.91 and a MAE of 1.41 were respectively observed. The RMSE and MAE values of Prodepth are also lower than those of the Yuan-Wang method and the Rdpred method, decreased by 0.17 and 0.13, 0.10 and 0.07, respectively.

**Table 5:** Prediction performance comparison of prediction methods based on the current dataset.

Methods	Dimensions of feature vectors	CC	RMSE	MAE
Yuan-Wang method	316	0.64	1.91	1.41
RDpred	368	0.68	1.84	1.36
Prodepth (this work)	435	0.71	1.74	1.28

These results indicate that Prodepth provides better prediction performance in comparison with the other two methods. Yuan and Wang' method utilized the PSI-BLAST scoring matrix and protein size information as the only input into the SVR predictors [36], while the RDpred method was based on the combination of PSI-BLAST profile and the predicted secondary structure information [37]. In contrast to the two methods, Prodepth not only utilized the PSI-BLAST profile and predicted secondary structure information, but also took into account other important local sequence and structural features and global features, particularly predicted solvent accessibility, natively unstructured region, percentage of exposed/buried residues, secondary structures and ordered/disordered residues, which might be the main reason that accounts for the improved prediction performance of using the Prodepth approach.

### Case study

**Figure 7:**
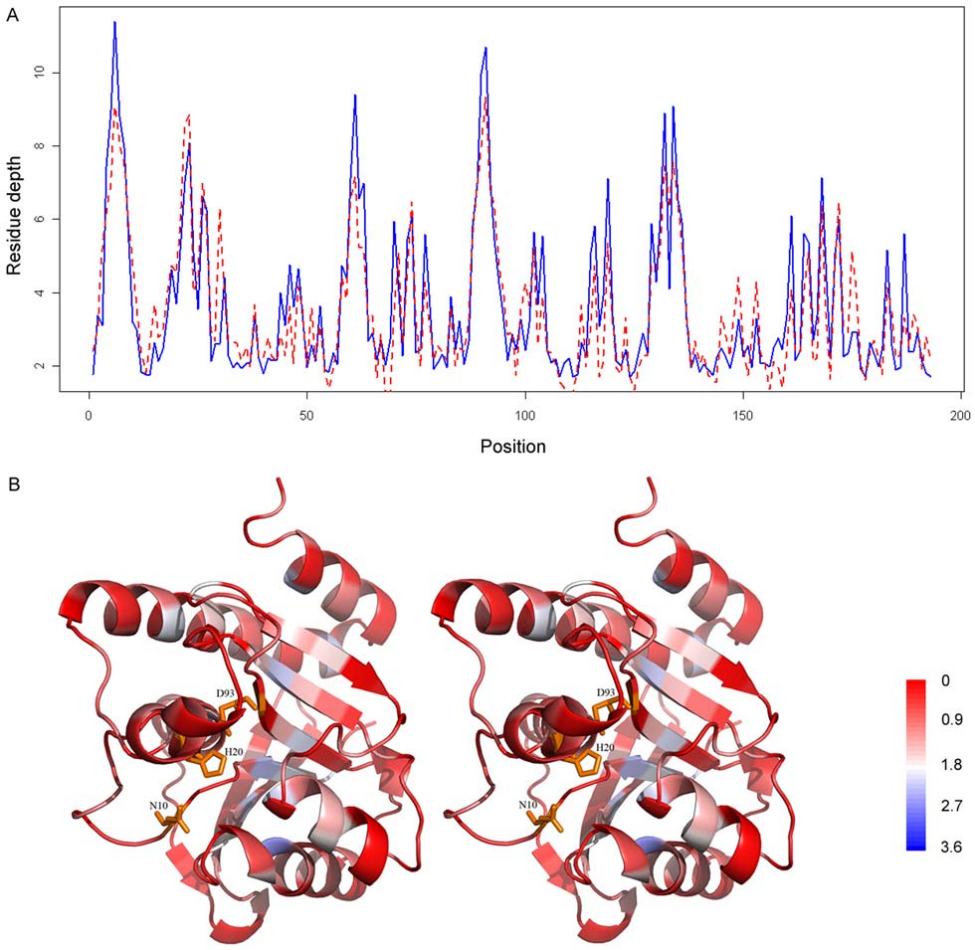
The predicted and observed residue depth profiles for the *Escherichia coli* peptidyl-tRNA hydrolase (PDB code: 2pth, chain A), as well as the structural mapping of the predicted RD profiles. In Figure 7A, the blue solid line represents the observed RD values, while the red dashed line represents the predicted RD values. In Figure 7B, the sequence regions predicted with different mean absolute errors are colored with a color scale going from red to blue, where red corresponds to the best predicted regions and blue to worst predicted regions. The active site residues (N10, H20 and D93) are highlighted by the orange sticks [51]. The structural images are prepared using the program PyMOL [81]. For the sake of visualization, structural figures are shown in stereo.

**Figure 8:**
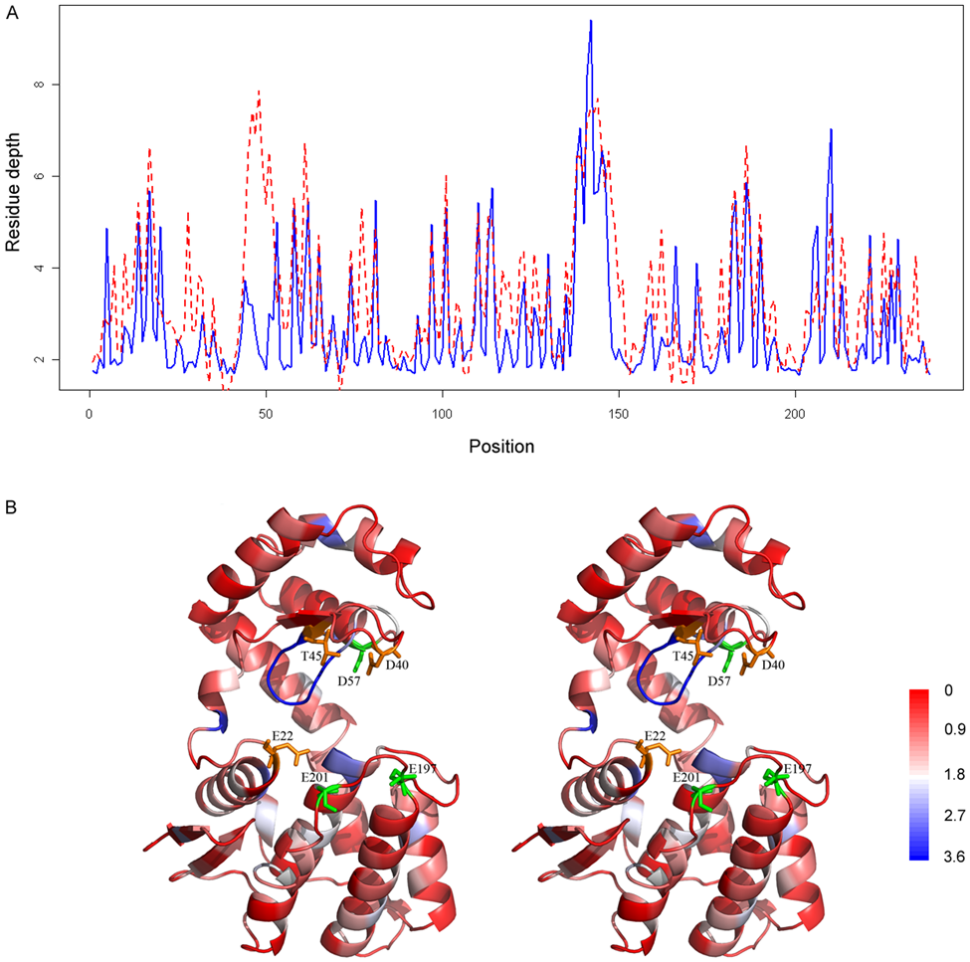
The predicted and observed residue depth profiles for the anti-fungal chitosanase (PDB code:1chk, chain A), as well as the structural mapping of the predicted RD profiles. In Figure 8A, the blue solid line represents the observed RD values, while the red dashed line represents the predicted RD values. In Figure 8B, the sequence regions predicted with different mean absolute errors are colored with a color scale going from red to blue, where red corresponds to the best predicted regions and blue to worst predicted regions. In addition, the active site residues (E22, D40 and T45) are highlighted by the orange sticks, while the functionally important residues involved in chitosan substrate binding (D57, E197 and E201) are represented by dark green sticks [52], [53]. The structural images are prepared using the program PyMOL [82]. For the sake of visualization, structural figures are shown in stereo.

We illustrated the performance of the Prodepth predictor by presenting two examples and showed their predicted RD profiles with the structural mapping of the MAE values on the three-dimensional structures in Figure 7 and 8. The first example is the *Escherichia coli* peptidyl-tRNA hydrolase (PDB code: 2pth, chain A) [51], which is well predicted with a CC of 0.89 and RMSE of 0.93. For the majority regions of this protein, there is a good agreement between the predicted and observed RD values despite that several separate residue positions such as 6, 61, 91, 132 and 134 are poorly predicted (blue), as can be seen from Figure 7A. Interestingly, many of these residues map to the hydrophobic core (Figure S3), however, it is unclear from sequence or structural perspective why these regions are poorly predicted.

The second protein is the anti-fungal chitosanase (PDB code: 1chk, chain A) [52], [53], for which RD is predicted with a CC of 0.71 and RMSE of 1.23. As can be seen from Figure 8, most of the RD values are well predicted. Interestingly, exceptions mainly map to the active site cleft. We argue this may be because the active site of an enzyme requires unusual properties with respect to the rest of the protein, for example, many active sites (including the current example provided here) comprise deeply protected clefts which may be required for interaction with substrates that include substantial hydrophobic patches. Regions from residue position 46 to 51 and from 156 to 161 were strongly over-predicted, while regions from position 138 to 142 are under-predicted. We can also see that a fragment of coiled residues are colored by blue, from their 3D structural mapping of the predicted RD profiles. It might be that these coiled residues are characterized by a variety of sequence features, which makes them less effectively represented in the available data and makes it more difficult for the trained models to grasp their specific roles.

### The Prodepth Webserver

The Prodepth webserver can be accessed at http://sunflower.kuicr.kyoto-u.ac.jp/~sjn/Prodepth/ for online prediction of RD from protein sequences only. It is developed to facilitate RD prediction analysis for sequences submitted by interested users. Moreover, aside from RD prediction, Prodepth can be used to predict the accessible surface area (ASA) value for each residue given the primary sequence only. Based on the predicted ASA and RD values, it will further output the two-state solvent accessibility prediction by classifying a residue as being exposed or buried.

The web interface is fairly straightforward to use: only the one-letter FASTA format of the sequence and the user's Email address need to be submitted. Then after the completion of the prediction task, user will receive an Email containing a link pointing to a temporary webpage that contains the detailed prediction results.

## Discussion

With the growth of sequenced data generated by the large-scale genomics projects, efficient and accurate structural determination and annotation has been the major focus of current structural genomics initiatives [54].This has created great demand for efficient computational approaches that help to narrow the sequence-structure gap. Machine learning techniques that cater for this demand are becoming attractive and have been successfully applied in many bioinformatics studies. However, due to the delicate sequence-structure relationship, predicting residue depth from protein primary sequences is an ongoing challenging task in structural bioinformatics [8], [36], [37]. In this paper, we presented a novel computational framework to accurately predict residue depth values from protein sequences only. We hope that this approach will add to the current efforts of sequence-based RD prediction.

Several factors can contribute to the improved prediction performance of our approach in predicting RD values from sequences alone. Firstly, since evolutionary information in the form of PSI-BLAST profiles has been demonstrated to have important influence on prediction accuracy, we incorporated this information into SVR in this study. Secondly, we added the important solvent accessibility predicted by SCRATCH and natively disordered region information by DISOPRED along with other global sequence features. Remarkably, we found that the use of predicted solvent accessibility information considerably improves the prediction accuracy, implying the important role solvent accessibility plays in predicting residue depth profiles. Thirdly, we systematically investigated several sequence encoding schemes to assess their respective prediction performance and the impacts of different sequence features on the prediction accuracy. Our predictions were evaluated by a 5-fold cross-validation approach based on a well-defined high-quality three-dimensional structure dataset. Among eight different sequence encoding schemes, the encoding scheme ‘ALL’ that incorporates all the relevant local and global sequence features, in conjunction with the predicted solvent accessibility and natively disordered regions outperforms other encoding schemes, with a CC of 0.71, a RMSE of 1.74 and a MAE of 1.28, respectively. The comparison indicates that our method could provide better prediction performance when compared with the other two methods.

Another important finding is that different secondary structures have different residue burial preferences in the protein structures. For instance, regular secondary structures such as α-helix, β-strand tend to be distributed with larger depth values, while non-regular secondary structures like coils are more inclined to be located with smaller depth values. These tendencies have different effects on the final prediction performance of RD, which has been taken into consideration in our SVR models. Furthermore, our method can be readily utilized to perform the conventional two-state (exposed/buried) prediction based on the real-valued prediction results of RD.

We further illustrated the prediction performance of the Prodepth server by highlighting two representative case studies (Figure 7 and 8), namely, the *Escherichia coli* peptidyl-tRNA hydrolase [51] and the anti-fungal chitosanase [52], [53]. In addition, as an implementation of our approach, we have developed a prediction webserver Prodepth to facilitate the RD prediction analysis for interested users. Prodepth can also provide an accurate prediction of accessible surface area (ASA), a traditional solvent exposure measure that provides important complementary information to RD.

In this study, we proposed a new approach to predict residue depth from primary sequences only, by combining a number of useful sequence and structural features including the PSI-BLAST profiles, predicted secondary structure, solvent accessibility information, natively disordered region, as well as some global sequence features. Comparison with the other two state-of-art methods illustrates the effectiveness of our approach. We hope that the developed Prodepth sever can become a powerful tool in sequence-based prediction of RD and ASA values and help towards the identification of functionally important residues, or key residues in the folding nucleus from protein primary sequences.

## Materials and Methods

### Dataset

The analysis is based on a representative dataset of high-quality protein three-dimensional structures, which was downloaded from the PDB-REPRDB server [55] that provides representative protein structures from PDB [56]. The dataset was originally prepared using the following criteria: all the structures in this dataset were determined using X-ray crystallography with resolution ≤2.0 Å, R-factor ≤0.25 and R-free factor ≤0.25; sequence length should be greater than 60 amino acid residues and without any chain breaks; each two sequences have sequence identity less than 30%. For certain PDB chains, the hsexpo progam [26] used to calculate the RD values had errors. Such erroneous PDB chains were thus discarded.

We further searched each PDB ID in the dataset and retrieved their SCOP superfamily classifications [57]. Those PDB chains without the SCOP superfamily annotations were not retained. After applying these procedures, we obtained a final dataset containing 489 protein chains (473 PDBs and totally 124,082 residues). The names of the protein chains, their protein sequences in FASTA format, the calculated RD values, and the corresponding SCOP superfamily classification were given in Dataset S1, S2 and S3, respectively. For an objective evaluation of the current approach, we performed a stringent 5-fold cross-validation test to examine the prediction performance. That is, the whole dataset was divided into 5 roughly equal subsets based on the SCOP superfamily-based criterion: no sequence(s) in the testing set should be in the same superfamily as another sequence(s) in the training set. This procedure was adopted to avoid the overestimation of prediction accuracy and reduce the impact of sequence or domain homology on the prediction performance [58].

### Accessible surface area (ASA)

The accessible surface areas for all residues in our dataset were calculated using the DSSP program [38]. Additionally, the absolute values were further divided by the maximum ASA values for the same residue type to obtain the relative accessible surface area (rASA) of each residue, as defined in Rost and Sander [6]. Secondary structure information was also annotated using the DSSP program [38].

### Residue depth (RD)

According to its definition, atom or residue depth can be computed as the distance between the residue of interest and its nearest neighboring water molecule or protein surface [7]–[9]. The detailed procedures to calculate residue depth in this study are described as follows. First, we calculate the accessible surface area for each atom and the whole molecule (the probing sphere radius is set as 1.4 Å). Second, atom depth is calculated as the distance between this atom and its nearest vertex. Third, a residue's RD is calculated as the average atom depth for all atoms except the hydrogen atoms in this residue. The hsexpo progam is used to calculate the RD values for all residues in a PDB file and the calculated results will be written out in this PDB file's B factor field [26]. Before input into the SVR, we normalize the RD values for all residues using 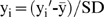, where *y_i_* is the normalized RD value of residue *i*, 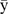 is the average RD value, and *SD* is the standard deviation. Thus, most of the normalized RD values could fall into the range of [0, 1], for the sake of data handling and the SVR input.

### Support vector regression (SVR)

Support vector machine is a sophisticated supervised machine learning technique that is built based on statistical learning theory [59], [60] and has been widely used in the applications of bioinformatics. Note that support vector machine (SVM) has two practical modes: support vector classification (SVC) and support vector regression (SVR). Particularly, in comparison with the SVC, the SVR has excellent regression abilities to infer the property values from a limited dataset of samples and it is especially effective when the input data is not linearly separable and the kernel function is required to map the data into a higher dimensional space to find the optimal separating hyperplane. Due to its regression advantages, its computing speed, its ability to control error, and as well as its superior performance over other machine learning techniques [59], [60], the SVR has been attracting more and more attention and has been successfully applied in predicting gene expression level [61], accessible surface area [62], residue contact number [40], missing value in microarray data [63], MHC-binding peptides [64], [65], residue-wise contact orders [41], disulfide connectivity [42] and half-sphere exposure [27], improving sequence alignment quality [66] and ranking predicted protein structures [67].

As we are more interested in predicting the RD values from protein sequences, we chose the SVR to train the predictive models and perform the prediction tasks. As the implementation of the SVR approach, the SVM_light package [68] (available at http://svmlight.joachims.org/) was employed. Particularly, we selected radial basis kernel function (RBF) at ε = 0.01, γ = 0.01 and *C* = 5.0 to build the SVR models. This combination of parameters has been shown to provide the best prediction performance in the preliminary analysis through selecting and comparing different combinations of *C* and ε and examining their respective prediction performances, using the PSI-BLAST profiles based on the five-fold cross-validation tests. In the following analysis, we then constantly set ε as 0.01, γ as 0.01 and *C* as 5.0 to evaluate the prediction performance of other sequence encoding schemes.

### Sequence encoding schemes

For a comprehensive investigation, we employed eight different encoding schemes in order to examine their corresponding influence on the prediction performance. These encoding schemes include both the global and local sequence features. With respect to the local sequence feature extraction, a sliding window method was used to capture the sequence environment [27], [42]. That is, we used the fixed window size of 15 residue centered on the residue of interest and then extracted the sequence profiles in terms of the PSSMs, PSIPRED-predicted secondary structure matrices, as well as the predicted solvent accessibility matrices (will be discussed in the following sections).

#### Position-specific scoring matrix (PSSM)

A residue's PSSM in the form of PSI-BLAST profile [69] contains important evolutionary information that determines whether this residue is conserved in its family of related proteins [70], [71]. Each element in the PSSM represents the likelihood of each residue position in the multiple sequence alignment of a protein class. PSSMs have been successfully applied in the prediction studies of many aspects in structural bioinformatics and have been shown to be helpful for improving the prediction performance [72], [73], [74], [75], [76], [77]. It is generally estimated that the incorporation of the PSSMs will lead to an increase of the overall prediction accuracy by 1–5% [45], [47]–[49]. Therefore, in this study, we queried each protein sequence in our dataset and extracted the PSSM profiles by running PSI-BLAST [69] against the NCBI nr database, in a standard manner (by three iterations, with a default cutoff *E*-value). All the elements in the PSSM were divided by 10 for normalization, so that most of the values were in the range of −1.0 and 1.0. For a given residue, its local sequence fragment was extracted and encoded as a 20×(2*l*+1)-dimensional vector using a sliding window scheme, where *l* denotes the half window size and 2*l*+1 is the full window length. In this study, we consistently fixed the window size at 15 (half window size *l* = 7), which has been suggested to lead to the overall best performance in previous studies [36], [37].

#### Predicted secondary structure (PSS)

The secondary structure information was predicted using the PSIPRED program developed by Jones [78]. PSIPRED is an accurate neural network-based predictor for the prediction of three-state (helix, strand and coil) secondary structure purpose with an accuracy of up to 80% [78]. In our previous studies, we have shown that using the PSIPRED-predicted secondary structure could significantly improve the prediction performance [27], [41], [42]. Similarly, for a given residue, its local three-state secondary structure profile was taken from a sliding window of 15 consecutive residues.

#### Predicted solvent accessibility (PSA)

The two-state solvent accessibility of each residue in the dataset was predicted using the SSpro program implemented in the SCRATCH package [50]. SSpro could predict the solvent accessibility status for each residue in a protein sequence, whose output result comes in a binary format- either as “exposed” or “buried”. Previous studies have indicated predicted solvent accessibility could be used to increase the accuracy and improve the reliability for predicting residue flexibility [13], natively unstructured regions [14] or loops [15], DNA-binding sites [16] and binding hotspots [17].

#### Predicted natively disordered region (DISO)

Natively unstructured/disordered region was predicted using the DISOPRED2 server [79], which is one of the leading servers for predicting natively disordered regions in proteins. As natively disordered regions are often functionally important and commonly associated with molecular assembly, protein modification and molecular recognition [80], [81], incorporating this information might be helpful for improving prediction performance. The probability of each residue being disordered generated by DISOPRED2 is used as the input to the SVR models.

#### Global sequence features

With regard to the global sequence features, we calculated the twenty amino acid compositions, the percentage of secondary structure classes, the percentage of exposed/buried residues, and the percentage of ordered/disordered residues. Additionally, the protein size descriptor based on protein molecular weight and protein sequence length were also utilized and normalized as the input into the SVR model. Incorporation of these global features has been shown to be helpful for improving the prediction performance in other groups' work [13]–[17].

### The Architecture of the Prodepth

**Figure 9:**
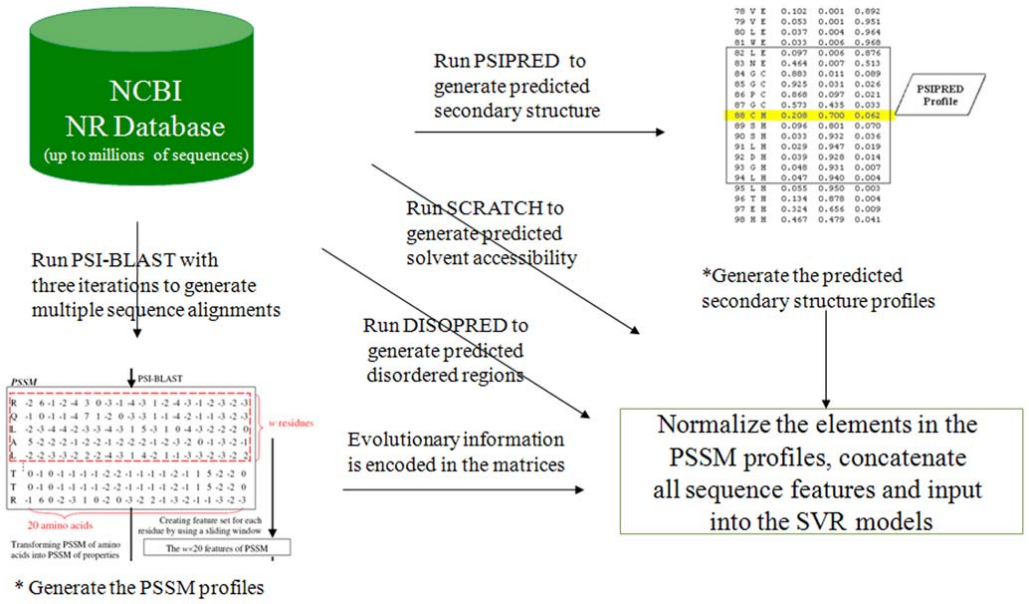
Generating local sequence input features using the PSI-BLAST, PSIPRED, SCRATCH and DISOPRED programs. See the main text for more details.

All the extracted local sequence profiles will be input into Prodepth along with other global sequence features. The procedures of generating local sequence input features for Prodepth is illustrated in Figure 9. Prodepth is comprised of three modules: the input, the prediction and the output module. First, users' submitted protein sequence in the FASTA format will be processed: PSI-BLAST, PSIPRED, SCRATCH and DISOPRED will be called to search this sequence against the non-redundant NCBI nr database, and the matrix profiles will be returned as the input to the prediction module.

### Performance assessment

For the performance evaluation of the real-value regression task in this study, the Pearson's correlation coefficient (CC) between the predicted and observed RD values is given by
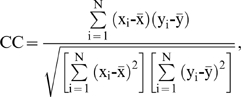
where x_i_ and y_i_ are the observed and predicted normalized RD values of the *i*-th residue, respectively, 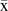 and 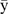 are their corresponding means and *N* is the total residue number in a protein sequence.

The root mean square error (RMSE) and mean absolute error (MAE) are respectively given by
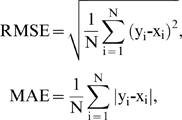
where x_i_ and y_i_ are the observed and predicted RD values of the *i*-th residue, respectively and *N* is the total residue numbers in a protein sequence.

In addition, we also calculate the R square values for each sequence encoding scheme used in this study, which is given by
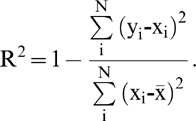
where *x_i_* and *y_i_* are the observed and predicted RD values of the *i*-th residue, respectively, 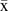 is the corresponding mean value and *N* is the total residue number in a protein sequence.

### Structural analysis

All structure images were rendered using the PyMOL program [82].

## Supporting Information

Dataset S1 List of 489 PDB structures used in this study. This file contains the PDB ID codes for the 489 protein structures compiled for the current study. Protein sequences in FASTA format and their respective chain names are also given.
(0.14 MB TXT)

Dataset S2 The calculated residue depth values for all residues in the dataset. The first and second columns are the residue name and the chain name of PDB structures, respectively. The third column corresponds to the original residue position in the PDB ATOM records, while the last column is the observed residue depth value.
(2.49 MB TXT)

Dataset S3 The 5-fold cross-validation list used in this study. 5-fold cross-validation test is performed to examine the prediction performance of the current approach: the whole dataset is randomly separated into 5 roughly equal subsets and at each cross-validation step, every subset is singled out as the testing set while the rest four subsets will be merged as the training set. For each protein sequence, its corresponding SCOP superfamily annotation is also provided.
(0.06 MB TXT)

Figure S1 Distributions of RD versus ASA based on the current dataset.
(1.11 MB TIF)

Figure S2 Consistency variation of the two-state solvent accessibility assignment based on residue depth and accessible surface area measures. The left y-axis denotes the ASA (red curve) value, while the right y-axis corresponds to the RD (blue curve) value.
(1.25 MB TIF)

Figure S3 Front view and back view of the Escherichia coli peptidyl-tRNA hydrolase (PDB code: 2pth, chain A) showing the hydrophobic core regions, as indicated by the dashed line circle.
(5.49 MB TIF)
