# Supplementary figures and images for: Prodepth: Predict Residue Depth by Support Vector Regression Approach from Protein Sequences Only
Source: PLoS One. 2009 Sep 17;4(9):e7072. doi: 10.1371/journal.pone.0007072 (PMC2742725; doi:10.1371/journal.pone.0007072)

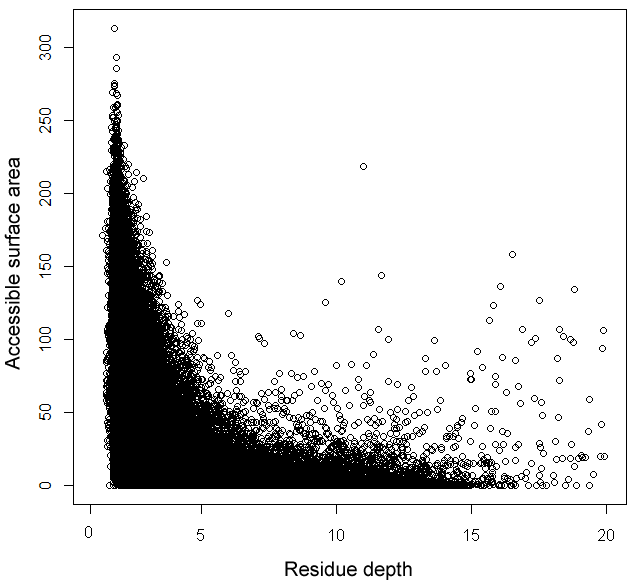

Supplement: Figure S1 — Distributions of RD versus ASA based on the current dataset. (1.11 MB TIF) [file pone.0007072.s004.tif]

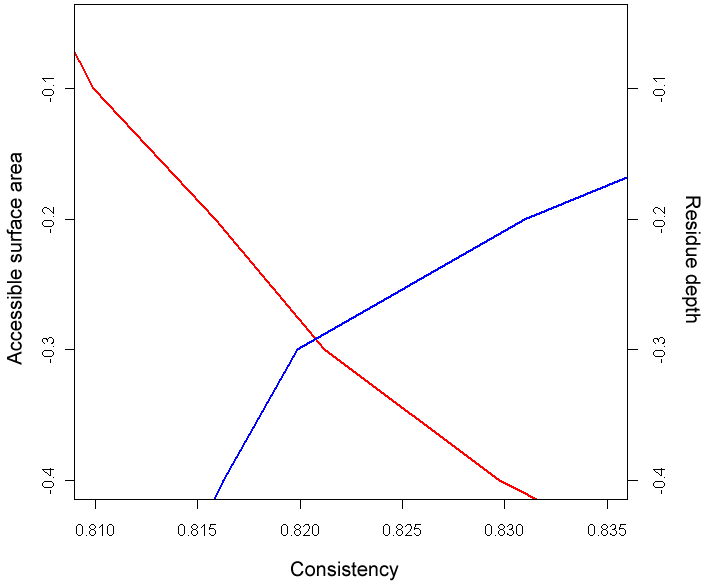

Supplement: Figure S2 — Consistency variation of the two-state solvent accessibility assignment based on residue depth and accessible surface area measures. The left y-axis denotes the ASA (red curve) value, while the right y-axis corresponds to the RD (blue curve) value. (1.25 MB TIF) [file pone.0007072.s005.tif]

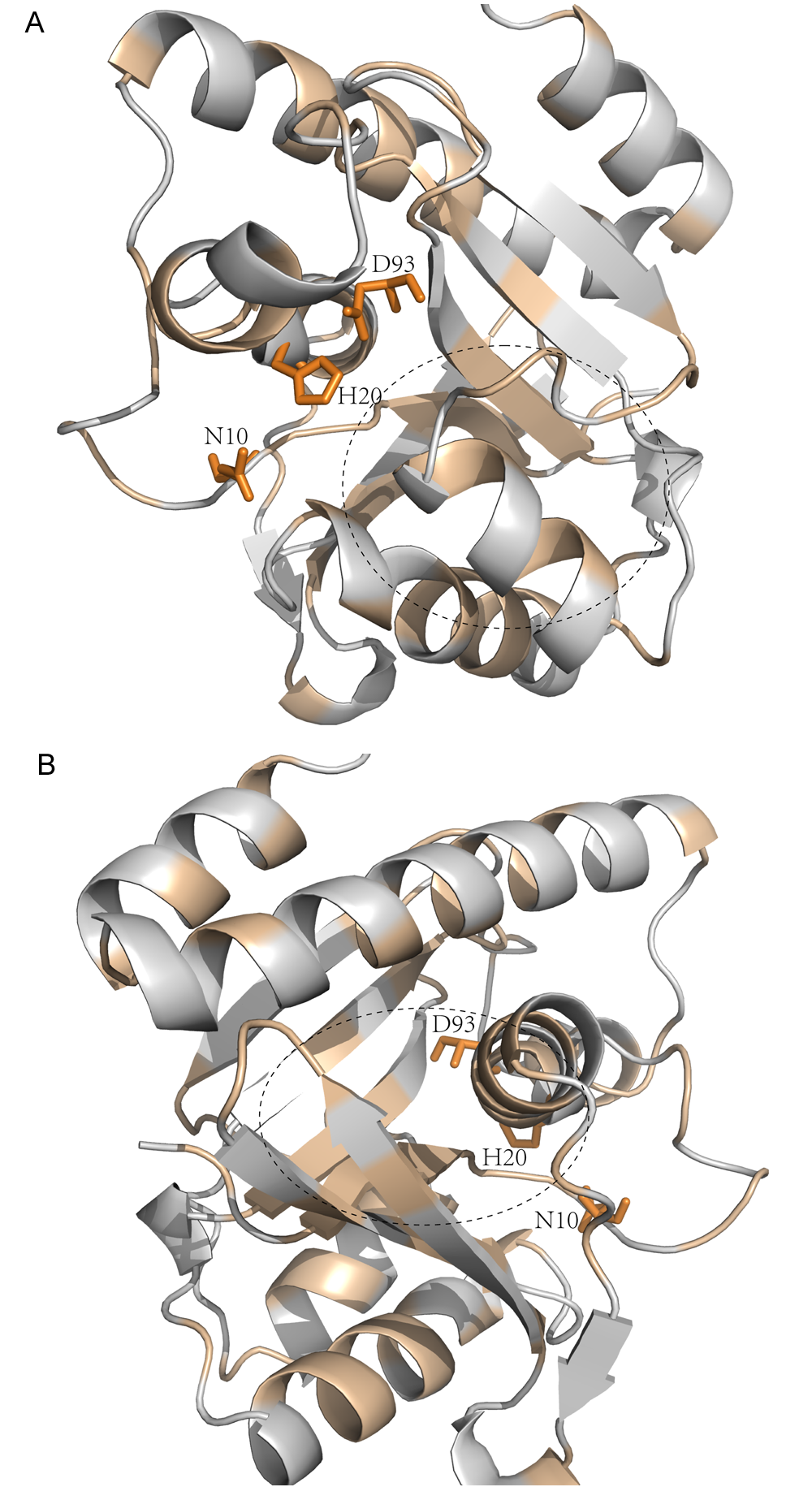

Supplement: Figure S3 — Front view and back view of the Escherichia coli peptidyl-tRNA hydrolase (PDB code: 2pth, chain A) showing the hydrophobic core regions, as indicated by the dashed line circle. (5.49 MB TIF) [file pone.0007072.s006.tif]
